# Leaf economics spectrum in rice: leaf anatomical, biochemical, and physiological trait trade-offs

**DOI:** 10.1093/jxb/ery322

**Published:** 2018-09-05

**Authors:** Dongliang Xiong, Jaume Flexas

**Affiliations:** 1 MARA Key Laboratory of Crop Ecophysiology and Farming System in the Middle Reaches of the Yangtze River, College of Plant Science and Technology, Huazhong Agricultural University, Wuhan, Hubei, China; 2 Centre for Carbon, Water and Food, University of Sydney, Brownlow Hill, New South Wales, Australia; 3 Research Group on Plant Biology under Mediterranean conditions, Instituto de Investigaciones Agroambientales y de Economía del Agua (INAGEA)–Universitat de les Illes Balears (UIB), Palma de Mallorca, Illes Balears, Spain

**Keywords:** Leaf economics spectrum, mesophyll conductance, mesophyll structure, nitrogen, photosynthesis, photosynthetic limitation

## Abstract

The leaf economics spectrum (LES) is an ecophysiological concept describing the trade-offs of leaf structural and physiological traits, and has been widely investigated on multiple scales. However, the effects of the breeding process on the LES in crops, as well as the mechanisms of the trait trade-offs underlying the LES, have not been thoroughly elucidated to date. In this study, a dataset that included leaf anatomical, biochemical, and functional traits was constructed to evaluate the trait covariations and trade-offs in domesticated species, namely rice (*Oryza* species). The slopes and intercepts of the major bivariate correlations of the leaf traits in rice were significantly different from the global LES dataset (Glopnet), which is based on multiple non-crop species in natural ecosystems, although the general patterns were similar. The photosynthetic traits responded differently to leaf structural and biochemical changes, and mesophyll conductance was the most sensitive to leaf nitrogen (N) status. A further analysis revealed that the relative limitation of mesophyll conductance declined with leaf N content; however, the limitation of the biochemistry increased relative to leaf N content. These findings indicate that breeding selection and high-resource agricultural environments lead crops to deviate from the leaf trait covariation in wild species, and future breeding to increase the photosynthesis of rice should primarily focus on improvement of the efficiency of photosynthetic enzymes.

## Introduction

The global covariation of leaf anatomical, biochemical, and gas exchange traits along with resource availability gradients, which is known as the leaf economics spectrum (LES), has received widespread attention (Wright *et al.*, 2004; Blonder *et al.*, 2011; Sack *et al.*, 2013, 2014; Niinemets, 2015; Onoda *et al.*, 2017). In brief, the global LES describes a continuum leaf spectrum, ranging from fast-growing species combined with low structural investment, high nutrient investment, quick return, and generally highly photosynthesizing leaves, to slow-growing species combined with high structure investment, low nutrient investment, slow return, and stress-tolerant leaves. The global LES is defined by several core leaf traits, including leaf mass per area (LMA), nitrogen (N) concentration (N_m_), the light-saturated photosynthetic rate per leaf mass (*A*_m_), and leaf lifespan (Wright *et al.*, 2004). However, the LES trait network is typically based on multiple non-crop species in natural ecosystems, and it is unclear whether the trade-offs among the leaf traits of domesticated species in agroecosystems are constrained by similar principles to those of non-crops. Agricultural environments often present strong ecological contrasts with the natural environments of non-crop species. This is because agriculture has historically been undertaken in resource-rich and low-risk areas, since farmers supply sufficient resources, including water and nutrients, and protect their crops from herbivores and pathogens (Meyer *et al.*, 2012; Zeder, 2015). A recent study confirmed that domestication has increased the leaf N and phosphorus (P) concentration by 57% in domesticated crops compared with their wild relatives (Delgado-Baquerizo *et al.*, 2016). Hence, it is logical to expect that selection for desired agronomic traits and breeding in resource-rich and predictable environments potentially shifts leaf trait correlations in domesticated crops from the LES correlations, which are based on non-crop species in natural ecosystems. A detailed investigation is required to fill important gaps in our understanding of the LES.

Despite broad recognition of the LES on multiple scales, the fundamental constraints underlying the LES are still unclear (Blonder *et al.*, 2011; Osnas *et al.*, 2013; Sack *et al.*, 2013; Niinemets, 2015; Onoda *et al.*, 2017). Several previous studies suggest that the core trait relationships of the LES are likely to operate via other traits (Shipley *et al.*, 2006; Sack *et al.*, 2013; Onoda *et al.*, 2017). For instance, Shipley *et al.* (2006) proposed that the ratio of the cell volume to cell wall volume was responsible for generating the LES. More recently, Onoda *et al.* (2017) highlighted the fundamental role of cell wall thickness and the proportion of N allocation to the cell wall in mediating the trait correlations in the LES. These studies basically focused on the role of N investment in the content of photosynthetic enzymes, especially Rubisco. However, in C_3_ plants, the area-based light-saturated photosynthetic rate (*A*) is limited by stomatal conductance (*g*_s_), mesophyll conductance to CO_2_ (*g*_m_), and/or the biochemistry of photosynthesis (Flexas, 2016). Under a given ambient condition, *g*_s_ relates to the leaf water status, which is largely determined by plant hydraulic conductance. Indeed, the coupling of LES traits and leaf hydraulic traits, including vein density (vein length per unit area; VLA) and leaf hydraulic conductance (*K*_leaf_), has been proposed (Blonder *et al.*, 2011; Sack *et al.*, 2013; Reich, 2014), while Li *et al.* (2015) observed that leaf vein traits are decoupled from LES traits. Recently, *g*_m_ was identified as an important photosynthetic limiting factor and was related to leaf structure and biochemical traits (Flexas *et al.*, 2012; Giuliani *et al.*, 2013; Tomás *et al.*, 2013; Xiong *et al.*, 2017*a*). Chloroplast size, number, and arrangement, mesophyll cell wall thickness (*T*_cw_), and the permeability of membranes are suggested as the major traits restricting *g*_m_. The biochemical limitations include the amount and activities of enzymes and metabolites involved in photosynthesis and the components of the thylakoid electron transport chains. Any change in N allocations within the leaf structure may potentially change the photosynthetic limitation processes and subsequently affect *A*.

Rice (*Oryza sativa*) is one of the most important crops worldwide, and enhancing *A* is considered a primary approach to improve grain yield (Long *et al.*, 2006; Zhu *et al.*, 2010; Long *et al.*, 2015). One ambitious approach is to convert current rice from C_3_ to C_4_ photosynthesis by introducing the CO_2_ concentrating mechanism (CCM) because the radiation use efficiency in C_4_ plants is higher than in C_3_ plants. However, introducing the CCM pathway requires many changes in both leaf anatomy and biochemical enzymes. In consideration of the elusive mechanistic basis of some of the photosynthetic traits and the methodological bottlenecks in certain aspects of biotechnology, simultaneous alterations of all of the limiting factors of photosynthesis, achieved by manipulating multiple genes, are unlikely to be accomplished in the near future (Zhu *et al.*, 2010; Flexas, 2016). Altering Rubisco kinetics is another approach for genetic engineering to improve the photosynthetic efficiency in crops. However, the complex assembly pathway of Rubisco and the apparent trade-offs in its kinetic parameters (Tcherkez *et al.*, 2006) indicate that creating a ‘better Rubisco’ is not likely, at least in the near future (Whitney *et al.*, 2011). Therefore, an efficient way to improve photosynthesis would be to exploit existing genetic variations in photosynthetic traits and the coordination among these traits in the existing genotypes. In fact, the genetic variations of leaf functional, anatomical, and biochemical traits have been observed in many crop species, including rice (Giuliani *et al.*, 2013; Gu *et al.*, 2014; Xiong *et al.*, 2017*a*). However, the coordination and/or trade-offs of intra-species leaf functional, anatomical, and biochemical traits (i.e. LES) has not been fully revealed.

In this study, we constructed a database that included leaf functional, anatomical, and biochemical traits of the most important cereal crop species, rice, to elucidate: (i) whether the concept of the LES can be applied within a domesticated crop and (ii) the roles of *g*_s_ and *g*_m_ in LES trait correlations. Based on these analyses, we then discuss the implications for improving photosynthesis and source use efficiency of rice along the LES.

## Materials and methods

**Fig. 1.**
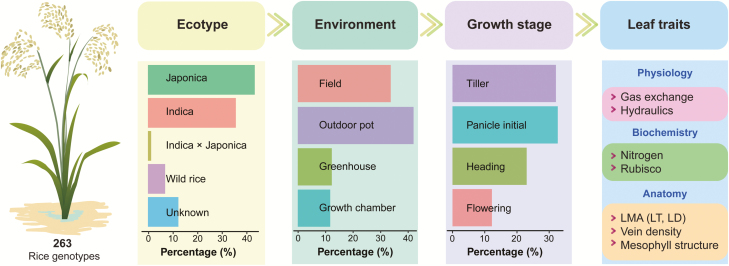
Summary of rice ecotypes, growth conditions (environment), plant developmental stage, and the leaf traits used for the analysis.

To test the correlations among the leaf traits, a database of rice leaf functional, biochemical, and structural traits of 263 genotypes growing in multiple conditions was compiled from the literature (see Supplementary data at *JXB* online). Because the aim of this study was to identify the potential effects of anatomical and biochemical traits on leaf function, the articles that reported one or more gas exchange traits and at least one structural or biochemical trait or leaf hydraulic conductance (*K*_leaf_) were included in the database. Leaf traits vary among plant species, growth conditions, and genotypes, and to extend the rice leaf spectrum as widely as possible, we included field, outdoor pot, greenhouse, and growth chamber studies (Fig. 1). Studies of short-term treatments, including light, CO_2_, temperature, and vapour-pressure deficit (VPD), were excluded. However, studies of long-term nutrient treatments were included in the database. In this study, we considered gas exchange parameters, including *A*, *g*_s_, and *g*_m_, and leaf anatomical and structural traits including the leaf mass per leaf area (LMA), leaf vein length per area (VLA), leaf thickness (LT), leaf density (LD), volume fraction of intercellular air space (*f*_IAS_), cell wall thickness (*T*_cw_), mesophyll surface area exposed to the intercellular air space per leaf area (*S*_m_), and mesophyll cell surface area occupied by chloroplasts exposed to the intercellular air space per leaf area (*S*_c_). The LD values were calculated as LMA/LT in the case of papers only reporting the LMA and LT values. The biochemical traits included the N content and Rubisco content. *K*_leaf_ was estimated using the evaporative flux method and its components, *K*_leaf_ inside the xylem (*K*_x_) and outside the xylem (*K*_ox_), were measured using the cutting method (e.g. Stiller *et al.*, 2003; Xiong *et al.*, 2017*a*). All of the data were extracted directly from the tables, text, and supplementary information in the original papers or indirectly from the figures, and all of the data were later converted to their standard units. Other information, if available, such as temperature, the maximum rate of carboxylation (*V*_cmax_), and the maximum rate of electron transport (*J*_max_), were also extracted for further analysis.

Many approaches have been developed to estimate *g*_m_. In the current database, three major common methods were used: (i) the online carbon isotope discrimination method (Evans *et al.*, 1986), (ii) the combined chlorophyll fluorescence and gas exchange method (Harley *et al.*, 1992), and (iii) the curve-fitting method (Ethier & Livingston, 2004). In all of the studies providing *g*_m_ values from the curve-fitting method, parallel estimates were provided by using the combined chlorophyll fluorescence and gas exchange method, and the *g*_m_ values from these two methods are quite similar in rice (Xiong *et al.*, 2015*b*) as well as across other species (Carriquí *et al.*, 2015). Thus, for these studies, only the *g*_m_ values from the combined chlorophyll fluorescence and gas exchange method were used to analyse the relationship of *g*_m_ with the other traits. Several studies have compared the *g*_m_ values from the online carbon isotope discrimination method and from the combined chlorophyll fluorescence and gas exchange method, and noted a remarkable similarity in the values of *g*_m_ from these two methods (Kodama *et al.*, 2011).

The effect of environmental factors (i.e. light, CO_2_, and temperature) on the instantaneous gas exchange has been verified by many studies (Bernacchi *et al.*, 2002; Yamori *et al.*, 2011; Walker *et al.*, 2013; Xiong *et al.*, 2015*a*). In the current database, the gas exchange measurements were performed under light-saturated conditions, and the light was not considered to affect the inter-study comparisons. Although almost all of the studies claimed that gas exchange measurements were performed under ambient CO_2_ conditions, the actual CO_2_ concentrations ranged between 350 and 420 ppm with a mean of 379 ppm (Supplementary Fig. S1). Overall, the first quartile (Q1) of the CO_2_ concentration was 374 ppm and the third quartile (Q3) was 391 ppm. This result indicated that across all of the studies, the CO_2_ concentrations in most of the studies were very similar (~380 ppm). Leaf temperature is another important factor influencing photosynthesis, and in the current database, the leaf temperature ranged from 25.0 to 30.6 °C, with an average of 28.2 °C. This temperature range may potentially affect the rice gas exchange. However, previous studies show the variations in photosynthesis in this temperature range are quite small and the photosynthetic optical temperature of rice is around 30 °C (Yamori *et al.*, 2011; Scafaro *et al.*, 2012; von Caemmerer & Evans, 2015).

The relationships between the leaf N content (N_a_) and the physiological traits (*A*, *g*_s_, *g*_m_, *V*_cmax_, and *J*_max_) were fitted by a logistic model (Sinclair & Horie, 1989; Rotundo & Cipriotti, 2017) as:

$$y=\alpha [\frac{2}{1+e^{-\beta ({\text{N}}_{a}-\gamma )}}-1]$$

where *y* represents *A*, *g*_s_, *g*_m_, *V*_cmax_, or *J*_max_, α is the asymptotic *y* at high leaf N content, β is the curvature of the response, and γ is the leaf N content at which *y* is zero. Next, the photosynthetic N use efficiency (PNUE) was calculated as:

$$\text{PNUE}=\frac{\partial A}{\partial {\text{N}}_{a}}$$

A photosynthetic limitation analysis is a helpful tool to quantify the relative limitation of *g*_s_, *g*_m_, and the photosynthetic biochemistry on *A* (Grassi & Magnani, 2005; Buckley & Diaz-Espejo, 2015), and it has been widely used recently, especially under stress conditions (Flexas *et al.*, 2009; Galle *et al.*, 2009; Tosens *et al.*, 2016; Wang *et al.*, 2018). In this study, the photosynthetic limitations, including the relative stomatal (*l*_s_), mesophyll (*l*_m_), and biochemical (*l*_b_) limitations at different N_a_, were calculated using fitted *A*, *g*_s_, *g*_m_, and *V*_cmax_ values according to Grassi and Magnani (2005):

$$l_{\text{s}}=\frac{g_{\text{t}}\text{/}g_{\text{sc}}\times \partial A\text{/}\partial C_{\text{c}}}{g_{\text{t}}+\partial A\text{/}\partial C_{\text{c}}}$$

$$l_{\text{m}}=\frac{g_{\text{t}}\text{/}g_{\text{m}}\times \partial A\text{/}\partial C_{\text{c}}}{g_{\text{t}}+\partial A\text{/}\partial C_{\text{c}}}$$

$$l_{\text{b}}=\frac{g_{\text{t}}}{g_{\text{t}}+\partial A\text{/}\partial C_{\text{c}}}$$

where *g*_t_ is the total conductance, which is calculated as:

$$g_{\text{t}}=\frac{1}{\frac{1}{g_{\text{s}}}+\frac{1}{g_{\text{m}}}}$$

*C*_c_ is the CO_2_ concentration in the chloroplasts, which is calculated as:

$$C_{\text{c}}=C_{\text{a}}-\frac{A}{g_{\text{t}}}$$

where *C*_a_ is the ambient CO_2_ concentration, and 400 µbar was used in this study.

The differences in the slope and intercept of the bivariate relationships between rice and the global dataset (Glopnet) were tested by using standardized major axis tests with the R package, SMATR 3v (Warton *et al.*, 2012). All of the analyses in this study were performed in R v3.4.4 (R Core Team, 2018).

## Results

### Variation of leaf traits in rice

Most of the leaf functional, biochemical, and anatomical traits of rice in the current dataset showed considerable variability (Fig. 1). *A* and *g*_s_ showed the widest variation, which was 59-fold between the highest and the lowest, and the cell wall thickness (*T*_cw_) showed the narrowest variation, which was only 1.9-fold between the highest and the lowest. *A* varied from 0.66 to 38.8 μmol m^−2^ s^−1^ with a median of 20.8 μmol m^−2^ s^−1^, N_a_ varied from 0.47 to 2.83 g m^−2^ with a median of 1.20 g m^−2^, and LMA varied from 24.6 to 73.8 g m^−2^ with a median of 48.7 g m^−2^. The variation in *g*_m_, the Rubisco concentration per leaf area, and the Rubisco concentration per leaf mass was more than 20-fold, and the variation in the mass-based light-saturated photosynthetic rate (*A*_m_), the mass-based stomatal conductance (*g*_sm_), the mass-based mesophyll conductance (*g*_mm_), and the leaf density was more than 10-fold.

### Coordination of leaf traits

**Fig. 2.**
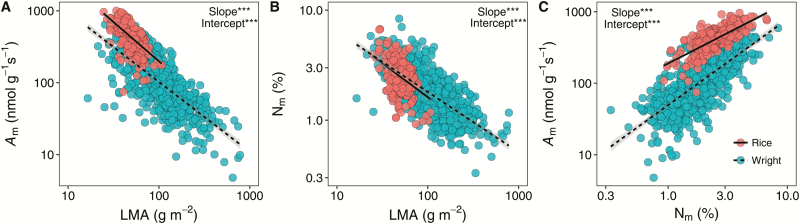
Relationships among the leaf nitrogen concentration (N_m_), the leaf mass per area (LMA), and the light-saturated photosynthetic rate per mass (*A*_m_). The circles represent data from Glopnet (Wright *et al.*, 2004) and rice as shown. The gray shaded area indicates the 95% confidence interval. Solid lines are standardized major axis (SMA) lines fitted to the rice dataset, and dashed lines SMA lines fitted to the Glopnet dataset. ***P < 0.001.

**Fig. 3.**
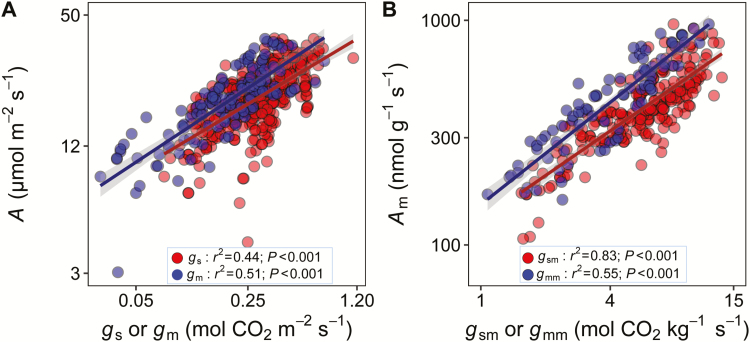
Correlations of the light-saturated photosynthetic rate (*A*) to the stomatal conductance (*g*_s_) and the mesophyll conductance (*g*_m_). (A) area-based correlations; (B) mass-based correlations. The gray shaded area indicates the 95% confidence interval.

**Fig. 4.**
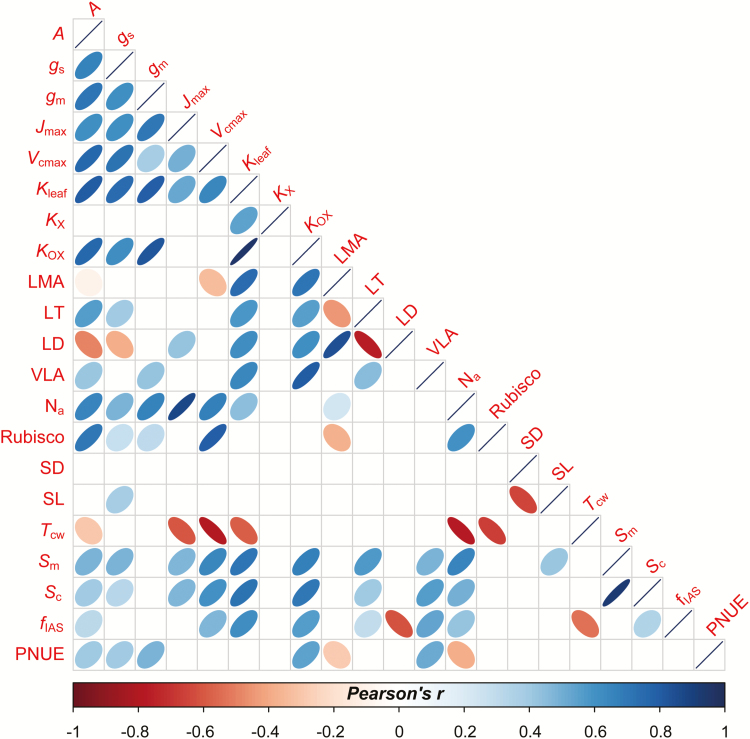
Correlations between the leaf traits (area base). The full name and units of the traits are shown in Supplementary Table S1. The correlations were estimated by the linear model and the 95% confidence level was used to draw the ellipses. The significant correlations are shown (*P*<0.05).

The leaf economic trait correlations in rice were observed in this study. As shown in Fig. 2, the bivariate correlations among LMA, *A*_m_, and N_m_ in the domesticated rice were generally consistent with the correlations for the natural species in Glopnet (Wright *et al.*, 2004). However, we observed significant differences in the slopes and intercepts of those correlations between rice and Glopnet (Fig. 2). Compared with the global database, rice tended to have a higher value of *A*_m_ at a given LMA or N_m_, but the decline in N_m_ with increasing LMA in rice was large. In rice, *A* was positively correlated with both *g*_s_ (*r*^2^=0.51, *P*<0.001) and *g*_m_ (*r*^2^=0.44, *P*<0.001; Fig. 3A), and the correlations were stronger when expressed on a mass basis (Fig. 3B). However, *A* (*r*^2^=0.03, *P*=0.43), *g*_s_ (*r*^2^=0.00, *P*=0.97), and *g*_m_ (*r*^2^=0.04, *P*=0.053) were independent of LMA in rice (Fig. 4; Supplementary Fig. S2). Otherwise, *A* and *g*_s_ increased with the leaf thickness, and decreased with leaf density. In contrast, *g*_m_ was independent of leaf thickness, as well as leaf density (Fig. 4). In this study, we also observed significant effects of the leaf anatomical and mesophyll structural traits on the leaf physiological traits (Fig. 4). *A* was positively correlated with the mesophyll (*S*_m_) and chloroplast (*S*_c_) surface exposed to the intercellular airspace but was negatively correlated with the cell wall thickness (*T*_cw_). We also found a significant effect of LMA, LT, and LD on leaf hydraulic conductance (*K*_leaf_). All of the estimated functional traits were independent of the leaf vein length per area (Fig. 4). Moreover, we observed significant interactions among the leaf structural traits (Fig. 4). For instance, the intercellular air space proportion (*f*_IAS_), *S*_m_, and *S*_c_ were positively correlated with LT, and *f*_IAS_ was negatively correlated with LD. In addition, *S*_m_ and *S*_c_ were tightly correlated.

**Fig. 5.**
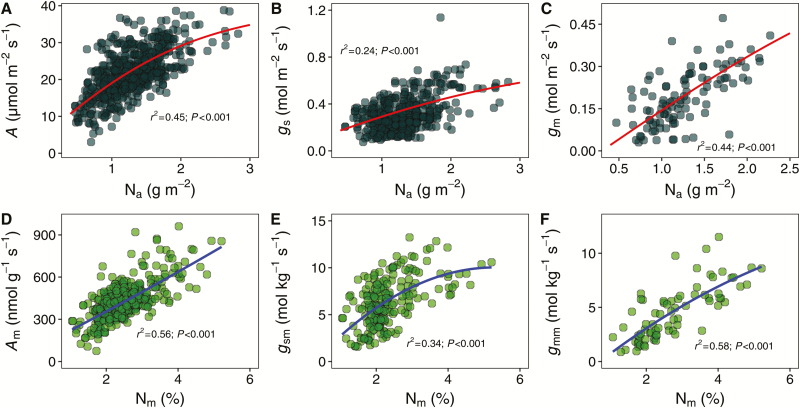
Effects of leaf nitrogen (N) concentration on the light-saturated photosynthetic rate (*A*) (A, D), the stomatal conductance (*g*_s_) (B, E), and the mesophyll conductance (*g*_m_) (C, F). (A–C) Area-based correlations; (D–F) mass-based correlations. The correlations were fitted by the logistic model as described in ‘Materials and methods’.

**Fig. 6.**
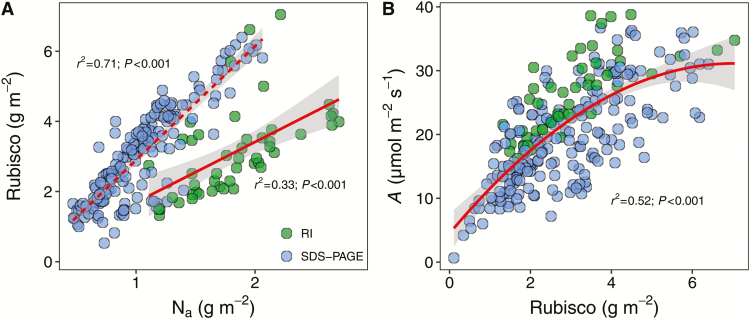
(A) Correlation between the N concentration per area (N_a_) and the Rubisco concentration, and (B) the correlation between the light-saturated photosynthetic rate (*A*) and the Rubisco concentration. RI, Rubisco concentration estimated by the radial immunodiffusion method; SDS-PAGE, Rubisco concentration estimated by SDS-PAGE. The gray shaded area indicates the 95% confidence interval.

The leaf N content was correlated with almost all of the leaf structural, functional, and biochemical traits (Fig. 4). The light-saturated photosynthetic rate (area base: *r*^2^=0.43, *P*<0.001; mass base: *r*^2^=0.50, *P*<0.001) the stomatal conductance (area base: *r*^2^=0.24, *P*<0.001; mass base: *r*^2^=0.34, *P*<0.001) and the mesophyll conductance (area base: *r*^2^=0.44, *P*<0.001; mass base: *r*^2^=0.58, *P*<0.001) were strongly correlated with the leaf N concentration on the area and mass base (Fig. 5). Basically, Rubisco concentration was linearly correlated with leaf N concentration, although the correlation differed strongly depending on the Rubisco estimation method (Fig. 6A). Moreover, *A* increased linearly with increasing Rubisco concentration at relatively low Rubisco concentrations and later levelled off (Fig. 6B). Typically, *A* (*r*^2^=0.65; *P*<0.001), *g*_s_ (*r*^2^=0.55; *P*<0.001), and *g*_m_ (*r*^2^=0.63; *P*<0.001) linearly increased with *K*_leaf_. However, the correlations were weaker for the mass than for the area base (Supplementary Fig. S3).

### Photosynthetic N use efficiency

**Fig. 7.**
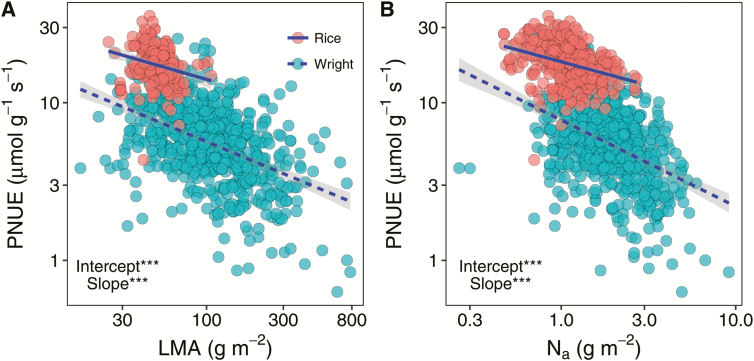
Effects of leaf mass per area (LMA) (A) and N concentration per leaf area (N_a_) (B) on photosynthetic N use efficiency (PNUE). The gray shaded area indicates the 95% confidence interval. Solid lines are standardized major axis (SMA) lines fitted to the rice dataset, and dashed lines SMA lines fitted to the Glopnet dataset. ***P < 0.001.

**Fig. 8.**
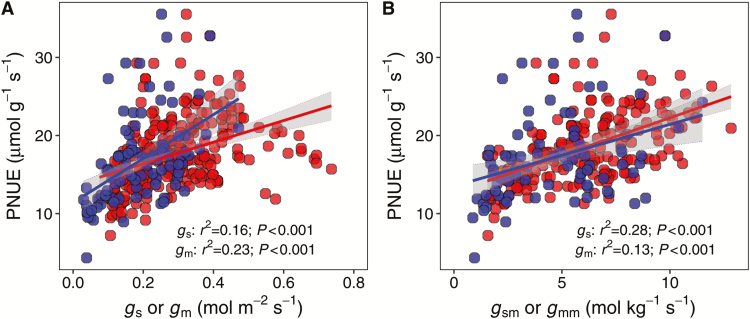
Contributions of the area- (A) and mass-based (B) stomatal conductance and mesophyll conductance to photosynthetic N use efficiency (PNUE). The gray shaded area indicates the 95% confidence interval.

The photosynthetic N use efficiency (PNUE) varied 10-fold across the database (Table 1). The PNUE was strongly correlated with the N_a_, VLA, and LMA but not with the other leaf anatomical and biochemical traits (Fig. 4). As shown in Fig. 6 and Supplementary Fig. S4, PNUE increased with *A* (*r*^2^=0.16, *P*<0.001) and *A*_m_ (*r*^2^=0.22, *P*<0.001) but decreased with LMA (*r*^2^=0.10, *P*<0.001) and N_a_ (*r*^2^=0.76, *P*<0.001). Compared with the global species database, rice tended to have a high PNUE under a given LMA, N_a_, N_m_, or *A*_m_ (Fig. 7; Supplementary Figs S4, S5). Moreover, as shown in Fig. 8, PNUE increased with increasing *g*_s_ (*r*^2^=0.16, *P*<0.001), *g*_sm_ (*r*^2^=0.28, *P*<0.001), *g*_m_ (*r*^2^=0.23, *P*<0.001), and *g*_mm_ (*r*^2^=0.13, *P*<0.001).

**Table 1.** Functional, biochemical, and anatomical trait variations in rice

Trait	Min.	Q1	Median	Mean	Q3	Max.
Function						
*A*	0.66	16.3	20.8	21.2	25.4	38.8
*g*_s_	0.075	0.219	0.290	0.313	0.383	1.138
*g*_m_	0.03	0.129	0.210	0.218	0.295	0.75
*K*_leaf_	3.31	4.99	7.20	7.77	10.72	13.46
*V*_cmax_	41.2	84.0	100.0	103.1	118.8	163.0
*J*_max_	59.4	111.4	140.9	149.3	170.3	308.3
*A*_m_	74.77	337.7	417.7	456.8	543.6	998.5
*g*_sm_	1.57	4.45	6.27	6.39	8.14	19.75
*g*_mm_	0.87	2.66	4.38	4.67	6.17	11.51
*K*_leafm_	0.122	0.135	0.199	0.199	0.263	0.307
Biochemistry						
N_a_	0.47	0.97	1.20	1.25	1.49	2.83
N_m_	0.92	1.94	2.24	2.47	2.81	6.73
Rubisco	0.11	1.74	2.68	2.90	3.74	7.05
Rubisco_m_	0.032	0.058	0.087	0.094	0.126	0.207
Anatomy						
LMA	24.6	42.8	48.7	49.2	56.1	73.8
VLA	2.98	3.98	4.34	4.62	5.28	6.73
VLA_m_	0.073	0.095	0.112	0.118	0.140	0.192
LT	0.058	0.084	0.132	0.148	0.216	0.282
LD	0.039	0.098	0.136	0.210	0.213	0.654
*f*_IAS_	7.7	16.7	19.5	19.3	23.1	28.8
*T*_cw_	0.125	0.155	0.167	0.168	0.183	0.235
*S*_m_	6.27	11.24	15.72	16.48	19.26	31.20
*S*_c_	6.43	11.54	15.72	15.81	18.04	30.59
Efficiency						
PNUE	4.33	14.52	17.31	18.12	21.1	44.4

The full name and units of the traits are shown in Supplementary Table S1. Min, minimum value; Q1, the first quartile; Q3, the third quartile; Max., Maximum value.

### Relative photosynthetic limitation

**Fig. 9.**
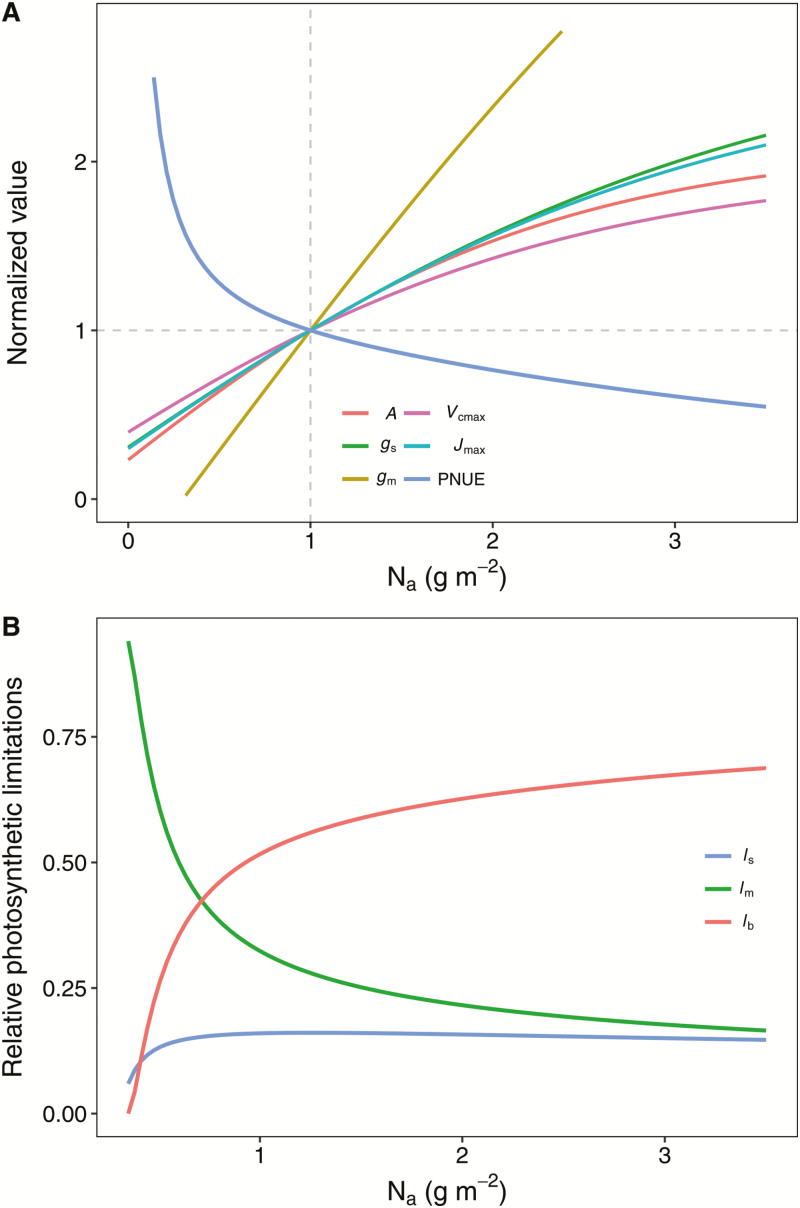
(A) Responses of light-saturated photosynthetic rate (*A*), stomatal conductance (*g*_s_), mesophyll conductance (*g*_m_), maximum carboxylation rate (*V*_camx_), maximum electron transport rate (*J*_max_), and photosynthetic N use efficiency (PNUE) to leaf N content per area (N_a_). (B) Changes in the relative photosynthetic limitation of stomatal conductance (*l*_s_), mesophyll conductance (*l*_m_), and biochemistry (*l*_b_) with N_a_. In (A), the parameters were normalized by dividing their values by 1.0 g m^−2^ of N_a_.

In this study, *A*, *g*_s_, *g*_m_, *V*_camx_, and *J*_max_ were modeled using the logistic model with the input of the leaf N content per area (N_a_), and the outputs shown in Table 2. The curvature (β) of the relationship between *A* and N_a_ was the highest, and the value of β of the relationship between *g*_m_ and N_a_ was the lowest. To estimate the sensitivity of the photosynthetic traits to N_a_, the values of *A*, *g*_s_, *g*_m_, *V*_camx_, and *J*_max_ were normalized by their values at 1.0 g m^−2^ of N_a_ (Fig. 9). The results showed that *g*_m_ was the most N-sensitive trait, and *V*_cmax_ was the most N-insensitive trait. We analysed the relative photosynthetic limitation based on the modeled photosynthetic traits. The leaf N status had a strong influence on the relative stomatal (*l*_s_), mesophyll (*l*_m_), and biochemical (*l*_b_), limitations in rice (Fig. 9). Overall, the biochemical limitation was the major photosynthetic limiting factor, which contributed more than 60% of the relative photosynthetic limitations, while *l*_s_ contributed than 10%. *l*_m_ strongly declined with N_a_, but *l*_b_ increased with N_a_.

**Table 2.** Fitted parameters relating light-saturated photosynthetic rate (*A*), stomatal conductance (*g*_s_), mesophyll conductance (*g*_m_), maximum carboxylation rate (*V*_cmax_), and maximum electron transport rate (*J*_max_) in responding to leaf N content per area (N_a_)

Trait	Fitted parameters			Value at N_a_=1.0
	α	β	γ	
*A*	40.04	0.8117	−0.2737	19.03
*g*_s_	0.8044	0.5350	−0.4183	0.2913
*g*_m_	0.8623	0.4813	0.3003	0.1438
*V*_cmax_	182.6	0.7052	−0.5763	92.17
*J*_max_	300.6	0.5600	−0.3988	116.7

## Discussion

### Leaf economics spectrum

For a given species, leaf traits vary depending on (i) the genotype, (ii) the growth environment and crop management, and (iii) the leaf and plant age (Wright *et al.*, 2004; Niinemets, 2015). In this study, 263 rice genotypes, which covered all of the ecotypes of rice growing under field, outdoor, greenhouse and/or growth chamber conditions, were included in our dataset (Fig. 1). Moreover, we also included multiple N management, growth stage, and leaf age variables. Considering that it would be almost impossible to estimate the leaf spectrum traits of all existing rice genotypes at all growth stages under all possible growth conditions, our dataset is a reasonable subset to represent the rice leaf spectrum.

Contrasting with two recent studies that indicated that the leaf trait correlations within a species may differ from the correlations among species (Anderegg *et al.*, 2018; Osnas *et al.*, 2018), the patterns of the leaf functional and anatomical trait correlations of rice were generally consistent with previously reported correlations in global (Wright *et al.*, 2004) and intraspecies LESs (Gagliardi *et al.*, 2015; Niinemets, 2015; Martin *et al.*, 2017). The significant trait covariations observed in this study suggested that the fundamental ecophysiological trade-off applies not only to natural species but also to domesticated species. This raises another question of whether human selection for traits in domesticated species has modified the leaf trait trade-offs from the global economic spectrum.

The light-saturated photosynthetic rate of domesticated rice was significantly higher than that in natural species in the Glopnet dataset at a given leaf N level, which indicated that the PNUE was improved by human selection in rice. In fact, an enhancement in *A* during breeding progress was observed in previous studies (Fischer *et al.*, 1998; Koester *et al.*, 2016). Although more information is needed to reveal the mechanism of PNUE enhancement in rice, greater N allocation to Rubisco may be one. The Rubisco concentration in rice is almost twice as high as in the natural species under a given leaf N level (Supplementary Fig. S6). A further difference from the Glopnet dataset was that both *A*_m_ and N_m_ decline faster with LMA in rice, suggesting that the influence of leaf structure on function were stronger in rice than in the native species in the Glopnet. Several recent studies demonstrated that correlations in the global LES arise, in part, by mathematical necessity (Lloyd *et al.*, 2013; Osnas *et al.*, 2013). However, these correlations reflect the physical effects of leaf structure on physiology (Sack *et al.*, 2013). In the current study, the different trait correlations between the two datasets was caused by biological factors because the same mathematical method was used. Moreover, the area-based bivariate correlations also support that the effects of leaf structure on physiological processes are stronger in rice (Fig. 4).

### Photosynthetic limitations in rice

The photosynthetic limitation factors in C_3_ plants have been widely investigated and three have been identified, namely *g*_s_, *g*_m_, and the biochemistry of photosynthesis, which includes the enzymes and metabolites involved in photosynthesis and the components of the thylakoid electron transport chain (Flexas, 2016). The positive relationships between photosynthetic rate and CO_2_ diffusion conductance, including the stomatal and mesophyll conductance, on both the area and mass base, indicated important limiting roles of *g*_s_ and *g*_m_ on rice photosynthesis (Fig. 3). Moreover, *A* was also tightly correlated with *V*_cmax_ and *J*_max_, which supports a biochemical limitation in photosynthesis. In fact, previous studies suggested that the major photosynthetic limiting factors in C_3_ plant leaves are modified by slight changes in the growing conditions (Yamori *et al.*, 2011; Wang *et al.*, 2018). Therefore, the LES trait trade-offs may relate to the photosynthetic limitations, shifting the cause to leaf anatomy and/or biochemical changes.

*g*_s_ is dependent on both the stomatal features, namely density and size and stomatal opening status. In our dataset, *g*_s_ of rice is independent of stomatal density and size (Fig. 4), which agreed with previous studies, suggesting that stomatal density and size mainly determine the maximum theoretical stomatal conductance, rather than the operational *g*_s_ (Franks & Beerling, 2009; Bartlett *et al.*, 2016; Xiong *et al.*, 2017*a*). The regulation of stomatal opening is complex, and many factors, including leaf water potential and abscisic acid concentration, are involved. In addition, under a given ambient condition, stable *g*_s_ is predominantly determined by the water supplement of the plant. In this study, the strong positive correlation between *g*_s_ and *K*_leaf_ supported a mechanistic relationship between the carbon assimilation and plant hydraulics (Brodribb *et al.*, 2005, 2007; Xiong *et al.*, 2015*b*, 2017*a*; Scoffoni *et al.*, 2016).

In C_3_ plants, photosynthesis and respiration primarily occur in the mesophyll; therefore, the trade-offs among the mesophyll structural, biochemical, and physiological traits are largely represented the LES (Onoda *et al.*, 2017). As one of the photosynthetic limiting factors, *g*_m_ is proposed to be related to both mesophyll structure and biochemistry (Evans *et al.*, 2009; Flexas *et al.*, 2012; Xiong *et al.*, 2015*a*, 2017*b*). Contrary to several previous studies (Tomás *et al.*, 2013; Tosens *et al.*, 2016; Xiong *et al.*, 2017*a*), *g*_m_ was independent of mesophyll structural traits in the current study. However, our results are consistent with the results obtained by Giuliani *et al.* (2013), who observed no correlation between *g*_m_ and mesophyll structural features across 24 genotypes of rice. Considering the important role of mesophyll structures in *g*_m_, the lack of correlation in this instance may be partly attributable to the narrow range of structural traits in the current dataset (Table 1). Our results indicated that *g*_m_ in rice might be predominately regulated by biochemical factors. In fact, *g*_m_ was positively correlated with N_a_, which is one of the most important leaf biochemical traits in determining leaf functions. However, a further analysis showed that the effects of N_a_ on *g*_m_ might be mediated by both biochemical factors and mesophyll structure. On one hand, a high N_a_ may promote aquaporin gene expression (Hacke *et al.*, 2010; Ding *et al.*, 2016) and the subsequent accumulation of aquaporins would enhance *g*_m_ by improving the permeability of biological membrane (Flexas *et al.*, 2006; Uehlein *et al.*, 2012; Mori *et al.*, 2014). Conversely, there were tight correlations between N_a_ and leaf structural traits, including *S*_c_ and *T*_cw_ (Fig. 4). A leaf with a high N_a_ tends to have a large size and/or number of chloroplasts, and hence a large *S*_m_ and *S*_c_ (Xiong *et al.*, 2015*a*). A negative relationship was observed between N_a_ and *T*_cw_ and may indicate that chloroplasts in leaves with low photosynthetic capacity may require thick and/or flexible cell walls to avoid excess light energy absorption, and the cell wall is known to restrict the diffusion of CO_2_ in the mesophyll.

According to the Farquhar–Berry–von Caemmerer model (Farquhar *et al.*, 1980), the biochemical limitation largely relates to the carboxylation capacity of Rubisco and the ribulose 1,5-bisphosphate regeneration rate, which is represented by *V*_cmax_ and *J*_max_, respectively. It is clear that *V*_cmax_ and *J*_max_ are highly dependent on the Calvin–Benson cycle and the amounts and activities of electron transport proteins. The allocation of leaf N to those photosynthetic proteins, especially Rubisco, is suggested to be determined by the species position in the LES (Onoda *et al.*, 2017). In this study, the Rubisco content per area increased linearly with N_a_ in rice, indicating a relatively constant proportion of N allocation to Rubisco (Fig. 6). However, the non-linear correlation between N_a_ and *V*_cmax_ indicated that Rubisco activity might vary with N_a_. Indeed, a reduction of Rubisco activation states (the ratio of the initial activity and the total activity) with an increase in N_a_ has been observed in previous studies (Cheng & Fuchigami, 2000; Warren & Adams, 2001). This result suggests that a *V*_cmax_ estimate based only on the Rubisco content (e.g. Buckley & Warren, 2014) may need to be calibrated by the Rubisco activation states. The mechanism of the low Rubisco activation state in the high N_a_ leaf is unclear, although the Rubisco activase activity, the ATP supplement capacity, and the storage function of Rubisco are suspected to cause the deactivation of Rubisco in high N leaves (Cheng & Fuchigami, 2000; Yamori *et al.*, 2006, 2012).

The photosynthetic limitation factors are apparently influenced by the leaf N concentration due to the profound effects of N on both leaf structural and leaf biochemical traits (Fig. 3). Therefore, the responses of the photosynthetic limitation factors to N_a_ may potentially exploit the mechanisms of the curvilinear correlation between the *A* and N_a_. In fact, *g*_s_, *g*_m_, and *V*_cmax_ responded differently to N_a_ in rice, and *g*_m_ was the most N-sensitive trait. The dramatic responses of *g*_m_ to N_a_ might be primarily caused by the enlargement of the chloroplasts and, to a lesser extent, the increasing *S*_c_ in the high N_a_ leaf (Xiong *et al.*, 2015*a*). More importantly, the relative limitation analysis suggested that *g*_m_ contributed the major relative limitation in *A* in the low N_a_ leaf. However, due to the dramatic increase in *g*_m_ with N_a_, photosynthetic biochemistry (i.e. *V*_cmax_ and *J*_max_) contributed the largest relative photosynthetic limitation in the high N_a_ leaf (Fig. 9). The results suggest that more attention should be paid to photosynthetic biochemistry for future *A*, as well as PNUE improvement in rice. However, we note that there are substantial variations in *g*_s_ and *g*_m_, and also biochemical limitations across genotypes, and therefore the photosynthetic limiting factors are actually genotype specific.

In summary, this study focused on the leaf structural, biochemical, and physiological trait variations and trade-offs in domesticated rice. The major bivariate correlations, including *A*_m_*vs.* N_m_, *A*_m_*vs.* LMA, and N_m_*vs.* LMA, of the LES traits in rice were shifted in comparison with the global LES dataset. *A*_m_ was higher in rice than in the natural species in the Glopnet at a given N_a_, and the breeding process in the past has therefore improved the PNUE of rice. The photosynthetic traits, including *g*_s_, *g*_m_, *V*_cmax_, and *J*_max_, were sensitive to the leaf structural and biochemical traits, and all of these traits increased with N_a_ in rice. Due to the asynchronous responses of the photosynthetic traits to the changes in N_a_, the major photosynthetic limitation steps were altered dramatically with N_a_, and biochemistry was the major limiting factor at an N_a_ above 1.0 g m^−2^. The leaf trait trade-offs underlying the general LES should be considered for future photosynthetic improvement in crops.

## Supplementary data

Supplementary data are available at *JXB* online.

Table S1. List of leaf traits in this study, and symbols and units adopted.

Fig. S1. Leaf temperature and the reference CO_2_ concentration inside the cuvette for gas exchange measurements.

Fig. S2. Influences of the leaf mass per area (LMA) on the light-saturated photosynthetic rate (*A*), the stomatal conductance (*g*_s_) and the mesophyll conductance (*g*_m_).

Fig. S3. Correlations of the leaf hydraulic conductance (*K*_leaf_) to the light-saturated photosynthetic rate (*A*), the stomatal conductance (*g*_s_), and the mesophyll conductance (*g*_m_) in rice.

Fig. S4. Correlations between the area-based light-saturated photosynthetic rate (*A*) and the photosynthetic N use efficiency (PNUE), and between the mass-based light-saturated photosynthetic rate (*A*_m_) and the PNUE.

Fig. S5. Effects of the N concentration per leaf mass (N_m_) and the mass-based light-saturated photosynthetic rate (*A*_m_) on the photosynthetic N use efficiency (PNUE).

Fig. S6. The correlation between Rubisco content and leaf N content within rice or among natural species (data from Onoda *et al.*, 2017).

Supplementary Material

## Data deposition

The data for the results presented here are available at the Dryad Digital Repository: https://doi.org/10.5061/dryad.6060q21 (Xiong and Flexas, 2018).

## References

[CIT0001] Anderegg LDL, Berner LT, Badgley G, Sethi ML, Law BE, HilleRisLambers J 2018 Within-species patterns challenge our understanding of the leaf economics spectrum. Ecology Letters 21, 734–744. 29569818 10.1111/ele.12945

[CIT0002] Bartlett MK, Klein T, Jansen S, Choat B, Sack L 2016 The correlations and sequence of plant stomatal, hydraulic, and wilting responses to drought. Proceedings of the National Academy of Sciences, USA 113, 13098–13103. 10.1073/pnas.1604088113 PMC5135344 27807136

[CIT0003] Bernacchi CJ, Portis AR, Nakano H, von Caemmerer S, Long SP 2002 Temperature response of mesophyll conductance. Implications for the determination of Rubisco enzyme kinetics and for limitations to photosynthesis in vivo. Plant Physiology 130, 1992–1998. 12481082 10.1104/pp.008250 PMC166710

[CIT0004] Blonder B, Violle C, Bentley LP, Enquist BJ 2011 Venation networks and the origin of the leaf economics spectrum. Ecology Letters 14, 91–100. 21073643 10.1111/j.1461-0248.2010.01554.x

[CIT0005] Brodribb TJ, Feild TS, Jordan GJ 2007 Leaf maximum photosynthetic rate and venation are linked by hydraulics. Plant Physiology 144, 1890–1898. 17556506 10.1104/pp.107.101352 PMC1949879

[CIT0006] Brodribb TJ, Holbrook NM, Zwieniecki MA, Palma B 2005 Leaf hydraulic capacity in ferns, conifers and angiosperms: impacts on photosynthetic maxima. New Phytologist 165, 839–846. 15720695 10.1111/j.1469-8137.2004.01259.x

[CIT0007] Buckley TN, Diaz-Espejo A 2015 Partitioning changes in photosynthetic rate into contributions from different variables. Plant, Cell & Environment 38, 1200–1211. 10.1111/pce.12459 25266511

[CIT0008] Buckley TN, Warren CR 2014 The role of mesophyll conductance in the economics of nitrogen and water use in photosynthesis. Photosynthesis Research 119, 77–88. 23609621 10.1007/s11120-013-9825-2

[CIT0009] Carriquí M, Cabrera HM, Conesa MÀ, et al 2015 Diffusional limitations explain the lower photosynthetic capacity of ferns as compared with angiosperms in a common garden study. Plant, Cell & Environment 38, 448–460. 10.1111/pce.12402 24995519

[CIT0010] Cheng L, Fuchigami LH 2000 Rubisco activation state decreases with increasing nitrogen content in apple leaves. Journal of Experimental Botany 51, 1687–1694. 11053458 10.1093/jexbot/51.351.1687

[CIT0011] Delgado-Baquerizo M, Reich PB, García-Palacios P, Milla R 2016 Biogeographic bases for a shift in crop C: N: P stoichiometries during domestication. Ecology Letters 19, 564–575. 26991808 10.1111/ele.12593

[CIT0012] Ding L, Gao L, Liu W, Wang M, Gu M, Ren B, Xu G, Shen Q, Guo S 2016 Aquaporin plays an important role in mediating chloroplastic CO_2_ concentration under high-N supply in rice (*Oryza sativa*) plants. Physiologia Plantarum 156, 215–226. 26382720 10.1111/ppl.12387

[CIT0013] Ethier GJ, Livingston NJ 2004 On the need to incorporate sensitivity to CO_2_ transfer conductance into the Farquhar–von Caemmerer–Berry leaf photosynthesis model. Plant, Cell & Environment 27, 137–153.

[CIT0014] Evans JR, Kaldenhoff R, Genty B, Terashima I 2009 Resistances along the CO_2_ diffusion pathway inside leaves. Journal of Experimental Botany 60, 2235–2248. 19395390 10.1093/jxb/erp117

[CIT0015] Evans JR, Sharkey TD, Berry JA, Farquhar GD 1986 Carbon isotope discrimination measured concurrently with gas exchange to investigate CO_2_ diffusion in leaves of higher plants. Functional Plant Biology 13, 281–292.

[CIT0016] Farquhar GD, von Caemmerer S, Berry JA 1980 A biochemical model of photosynthetic CO_2_ assimilation in leaves of C_3_ species. Planta 149, 78–90. 24306196 10.1007/BF00386231

[CIT0017] Fischer RA, Rees D, Sayre KD, Lu ZM, Condon AG, Saavedra AL 1998 Wheat yield progress associated with higher stomatal conductance and photosynthetic rate, and cooler canopies. Crop Science 38, 1467–1475.

[CIT0018] Flexas J 2016 Genetic improvement of leaf photosynthesis and intrinsic water use efficiency in C_3_ plants: Why so much little success?Plant Science 251, 155–161. 27593473 10.1016/j.plantsci.2016.05.002

[CIT0019] Flexas J, Barbour MM, Brendel O, et al 2012 Mesophyll diffusion conductance to CO_2_: an unappreciated central player in photosynthesis. Plant Science 193–194, 70–84. 10.1016/j.plantsci.2012.05.009 22794920

[CIT0020] Flexas J, Barón M, Bota J, et al 2009 Photosynthesis limitations during water stress acclimation and recovery in the drought-adapted *Vitis* hybrid Richter-110 (*V. berlandieri×**V. rupestris*). Journal of Experimental Botany 60, 2361–2377. 19351904 10.1093/jxb/erp069

[CIT0021] Flexas J, Ribas-Carbó M, Hanson DT, Bota J, Otto B, Cifre J, McDowell N, Medrano H, Kaldenhoff R 2006 Tobacco aquaporin NtAQP1 is involved in mesophyll conductance to CO_2_ in vivo. The Plant Journal 48, 427–439. 17010114 10.1111/j.1365-313X.2006.02879.x

[CIT0022] Franks PJ, Beerling DJ 2009 Maximum leaf conductance driven by CO_2_ effects on stomatal size and density over geologic time. Proceedings of the National Academy of Sciences, USA 106, 10343–10347. 10.1073/pnas.0904209106 PMC2693183 19506250

[CIT0023] Gagliardi S, Martin AR, Filho EDMV, Rapidel B, Isaac ME 2015 Intraspecific leaf economic trait variation partially explains coffee performance across agroforestry management regimes. Agriculture, Ecosystems & Environment 200, 151–160.

[CIT0024] Galle A, Florez-Sarasa I, Tomas M, Pou A, Medrano H, Ribas-Carbo M, Flexas J 2009 The role of mesophyll conductance during water stress and recovery in tobacco (*Nicotiana sylvestris*): acclimation or limitation?Journal of Experimental Botany 60, 2379–2390. 19321646 10.1093/jxb/erp071

[CIT0025] Giuliani R, Koteyeva N, Voznesenskaya E, Evans MA, Cousins AB, Edwards GE 2013 Coordination of leaf photosynthesis, transpiration, and structural traits in rice and wild relatives (genus *Oryza*). Plant Physiology 162, 1632–1651. 23669746 10.1104/pp.113.217497 PMC3707562

[CIT0026] Grassi G, Magnani F 2005 Stomatal, mesophyll conductance and biochemical limitations to photosynthesis as affected by drought and leaf ontogeny in ash and oak trees. Plant, Cell & Environment 28, 834–849.

[CIT0027] Gu J, Yin X, Stomph TJ, Struik PC 2014 Can exploiting natural genetic variation in leaf photosynthesis contribute to increasing rice productivity? A simulation analysis. Plant, Cell & Environment 37, 22–34. 10.1111/pce.12173 23937619

[CIT0028] Hacke UG, Plavcová L, Almeida-Rodriguez A, King-Jones S, Zhou W, Cooke JE 2010 Influence of nitrogen fertilization on xylem traits and aquaporin expression in stems of hybrid poplar. Tree Physiology 30, 1016–1025. 20610665 10.1093/treephys/tpq058

[CIT0029] Harley PC, Loreto F, Di Marco G, Sharkey TD 1992 Theoretical considerations when estimating the mesophyll conductance to CO_2_ flux by analysis of the response of photosynthesis to CO_2_. Plant Physiology 98, 1429–1436. 16668811 10.1104/pp.98.4.1429 PMC1080368

[CIT0030] Kodama N, Cousins A, Tu KP, Barbour MM 2011 Spatial variation in photosynthetic CO_2_ carbon and oxygen isotope discrimination along leaves of the monocot triticale (*Triticum× Secale*) relates to mesophyll conductance and the Péclet effect. Plant, Cell & Environment 34, 1548–1562. 10.1111/j.1365-3040.2011.02352.x 21707646

[CIT0031] Koester RP, Nohl BM, Diers BW, Ainsworth EA 2016 Has photosynthetic capacity increased with 80 years of soybean breeding? An examination of historical soybean cultivars. Plant, Cell & Environment 39, 1058–1067. 10.1111/pce.12675 26565891

[CIT0032] Li L, McCormack ML, Ma C, Kong D, Zhang Q, Chen X, Zeng H, Niinemets Ü, Guo D 2015 Leaf economics and hydraulic traits are decoupled in five species-rich tropical-subtropical forests. Ecology Letters 18, 899–906. 26108338 10.1111/ele.12466

[CIT0033] Lloyd J, Bloomfield K, Domingues TF, Farquhar GD 2013 Photosynthetically relevant foliar traits correlating better on a mass vs an area basis: of ecophysiological relevance or just a case of mathematical imperatives and statistical quicksand?New Phytologist 199, 311–321. 23621613 10.1111/nph.12281

[CIT0034] Long SP, Marshall-Colon A, Zhu XG 2015 Meeting the global food demand of the future by engineering crop photosynthesis and yield potential. Cell 161, 56–66. 25815985 10.1016/j.cell.2015.03.019

[CIT0035] Long SP, Zhu XG, Naidu SL, Ort DR 2006 Can improvement in photosynthesis increase crop yields?Plant, Cell & Environment 29, 315–330. 10.1111/j.1365-3040.2005.01493.x 17080588

[CIT0036] Martin AR, Rapidel B, Roupsard O, Van den Meersche K, de Melo Virginio Filho E, Barrios M, Isaac ME, Barton K 2017 Intraspecific trait variation across multiple scales: the leaf economics spectrum in coffee. Functional Ecology 31, 604–612.

[CIT0037] Meyer RS, DuVal AE, Jensen HR 2012 Patterns and processes in crop domestication: an historical review and quantitative analysis of 203 global food crops. New Phytologist 196, 29–48. 22889076 10.1111/j.1469-8137.2012.04253.x

[CIT0038] Mori IC, Rhee J, Shibasaka M, Sasano S, Kaneko T, Horie T, Katsuhara M 2014 CO_2_ transport by PIP2 aquaporins of barley. Plant & Cell Physiology 55, 251–257. 24406630 10.1093/pcp/pcu003 PMC3913445

[CIT0039] Niinemets Ü 2015 Is there a species spectrum within the world-wide leaf economics spectrum? Major variations in leaf functional traits in the Mediterranean sclerophyll *Quercus ilex*. New Phytologist 205, 79–96. 25580487 10.1111/nph.13001

[CIT0040] Onoda Y, Wright IJ, Evans JR, Hikosaka K, Kitajima K, Niinemets Ü, Poorter H, Tosens T, Westoby M 2017 Physiological and structural tradeoffs underlying the leaf economics spectrum. New Phytologist 214, 1447–1463. 28295374 10.1111/nph.14496

[CIT0041] Osnas JLD, Katabuchi M, Kitajima K, Wright SJ, Reich PB, Van Bael SA, Kraft NJB, Samaniego MJ, Pacala SW, Lichstein JW 2018 Divergent drivers of leaf trait variation within species, among species, and among functional groups. Proceedings of the National Academy of Sciences, USA 115, 5480–5485. 10.1073/pnas.1803989115 PMC6003520 29724857

[CIT0042] Osnas JLD, Lichstein JW, Reich PB, Pacala SW 2013 Global leaf trait relationships: mass, area, and the leaf economics spectrum. Science 340, 741–744. 23539179 10.1126/science.1231574

[CIT0043] **R Core Team** 2018 R: A Language and Environment for Statistical Computing. Vienna: R Foundation for Statistical Computing https://www.R-project.org/.

[CIT0044] Reich PB 2014 The world-wide ‘fast–slow’ plant economics spectrum: a traits manifesto. Journal of Ecology 102, 275–301.

[CIT0045] Rotundo JL, Cipriotti PA 2017 Biological limits on nitrogen use for plant photosynthesis: a quantitative revision comparing cultivated and wild species. New Phytologist 214, 120–131. 27943369 10.1111/nph.14363

[CIT0046] Sack L, Scoffoni C, John GP, Poorter H, Mason CM, Mendez-Alonzo R, Donovan LA 2013 How do leaf veins influence the worldwide leaf economic spectrum? Review and synthesis. Journal of Experimental Botany 64, 4053–4080. 24123455 10.1093/jxb/ert316

[CIT0047] Sack L, Scoffoni C, John GP, Poorter H, Mason CM, Mendez-Alonzo R, Donovan LA 2014 Leaf mass per area is independent of vein length per area: avoiding pitfalls when modelling phenotypic integration (reply to Blonder *et al*. 2014). Journal of Experimental Botany 65, 5115–5123. 25118296 10.1093/jxb/eru305 PMC4157720

[CIT0048] Scafaro AP, Yamori W, Carmo-Silva AE, Salvucci ME, von Caemmerer S, Atwell BJ 2012 Rubisco activity is associated with photosynthetic thermotolerance in a wild rice (*Oryza meridionalis*). Physiologia Plantarum 146, 99–109. 22324885 10.1111/j.1399-3054.2012.01597.x

[CIT0049] Scoffoni C, Chatelet DS, Pasquet-Kok J, Rawls M, Donoghue MJ, Edwards EJ, Sack L 2016 Hydraulic basis for the evolution of photosynthetic productivity. Nature Plants 2, 16072. 27255836 10.1038/nplants.2016.72

[CIT0050] Shipley B, Lechowicz MJ, Wright I, Reich PB 2006 Fundamental trade-offs generating the worldwide leaf economics spectrum. Ecology 87, 535–541. 16602282 10.1890/05-1051

[CIT0051] Sinclair TR, Horie T 1989 Leaf nitrogen, photosynthesis, and crop radiation use efficiency: a review. Crop Science 29, 90–98.

[CIT0052] Stiller V, Lafitte HR, Sperry JS 2003 Hydraulic properties of rice and the response of gas exchange to water stress. Plant Physiology 132, 1698–1706. 12857848 10.1104/pp.102.019851 PMC167106

[CIT0053] Tcherkez GG, Farquhar GD, Andrews TJ 2006 Despite slow catalysis and confused substrate specificity, all ribulose bisphosphate carboxylases may be nearly perfectly optimized. Proceedings of the National Academy of Sciences, USA 103, 7246–7251. 10.1073/pnas.0600605103 PMC1464328 16641091

[CIT0054] Tomás M, Flexas J, Copolovici L, Galmés J, Hallik L, Medrano H, Ribas-Carbó M, Tosens T, Vislap V, Niinemets Ü 2013 Importance of leaf anatomy in determining mesophyll diffusion conductance to CO_2_ across species: quantitative limitations and scaling up by models. Journal of Experimental Botany 64, 2269–2281. 23564954 10.1093/jxb/ert086 PMC3654418

[CIT0055] Tosens T, Nishida K, Gago J, et al 2016 The photosynthetic capacity in 35 ferns and fern allies: mesophyll CO_2_ diffusion as a key trait. New Phytologist 209, 1576–1590. 26508678 10.1111/nph.13719

[CIT0056] Uehlein N, Sperling H, Heckwolf M, Kaldenhoff R 2012 The *Arabidopsis* aquaporin PIP1;2 rules cellular CO_2_ uptake. Plant, Cell & Environment 35, 1077–1083. 10.1111/j.1365-3040.2011.02473.x 22150826

[CIT0057] von Caemmerer S, Evans JR 2015 Temperature responses of mesophyll conductance differ greatly between species. Plant, Cell & Environment 38, 629–637. 10.1111/pce.12449 25224884

[CIT0058] Walker B, Ariza LS, Kaines S, Badger MR, Cousins AB 2013 Temperature response of in vivo Rubisco kinetics and mesophyll conductance in *Arabidopsis thaliana*: comparisons to *Nicotiana tabacum*. Plant, Cell & Environment 36, 2108–2119. 10.1111/pce.12166 23869820

[CIT0059] Wang X, Wang W, Huang J, Peng S, Xiong D 2018 Diffusional conductance to CO_2_ is the key limitation to photosynthesis in salt-stressed leaves of rice (*Oryza sativa*). Physiologia Plantarum 163, 45–58. 29055043 10.1111/ppl.12653

[CIT0060] Warren CR, Adams MA 2001 Distribution of N, Rubisco and photosynthesis in *Pinus pinaster* and acclimation to light. Plant, Cell & Environment 24, 597–609.

[CIT0061] Warton DI, Duursma RA, Falster DS, Taskinen S 2012 smatr 3 – an R package for estimation and inference about allometric lines. Methods in Ecology and Evolution 3, 257–259.

[CIT0062] Whitney SM, Houtz RL, Alonso H 2011 Advancing our understanding and capacity to engineer nature’s CO_2_-sequestering enzyme, Rubisco. Plant Physiology 155, 27–35. 20974895 10.1104/pp.110.164814 PMC3075749

[CIT0063] Wright IJ, Reich PB, Westoby M, et al 2004 The worldwide leaf economics spectrum. Nature 428, 821–827. 15103368 10.1038/nature02403

[CIT0064] Xiong D, Flexas J 2018 Data from: Leaf economics spectrum in rice: leaf anatomical, biochemical and physiological trait trade-offs. Dryad Digital Repository, 10.5061/dryad.6060q21. PMC6255696 30189099

[CIT0065] Xiong D, Flexas J, Yu T, Peng S, Huang J 2017a Leaf anatomy mediates coordination of leaf hydraulic conductance and mesophyll conductance to CO_2_ in *Oryza*. New Phytologist 213, 572–583. 27653809 10.1111/nph.14186

[CIT0066] Xiong D, Huang J, Peng S, Li Y 2017b A few enlarged chloroplasts are less efficient in photosynthesis than a large population of small chloroplasts in *Arabidopsis thaliana*. Scientific Reports 7, 5782. 28720786 10.1038/s41598-017-06460-0 PMC5515944

[CIT0067] Xiong D, Liu X, Liu L, Douthe C, Li Y, Peng S, Huang J 2015a Rapid responses of mesophyll conductance to changes of CO_2_ concentration, temperature and irradiance are affected by N supplements in rice. Plant, Cell & Environment 38, 2541–2550. 10.1111/pce.12558 25923314

[CIT0068] Xiong D, Yu T, Zhang T, Li Y, Peng S, Huang J 2015b Leaf hydraulic conductance is coordinated with leaf morpho-anatomical traits and nitrogen status in the genus *Oryza*. Journal of Experimental Botany 66, 741–748. 25429002 10.1093/jxb/eru434 PMC4321541

[CIT0069] Yamori W, Masumoto C, Fukayama H, Makino A 2012 Rubisco activase is a key regulator of non-steady-state photosynthesis at any leaf temperature and, to a lesser extent, of steady-state photosynthesis at high temperature. The Plant Journal 71, 871–880. 22563799 10.1111/j.1365-313X.2012.05041.x

[CIT0070] Yamori W, Nagai T, Makino A 2011 The rate-limiting step for CO_2_ assimilation at different temperatures is influenced by the leaf nitrogen content in several C_3_ crop species. Plant, Cell & Environment 34, 764–777. 10.1111/j.1365-3040.2011.02280.x 21241332

[CIT0071] Yamori W, Suzuki K, Noguchi K, Nakai M, Terashima I 2006 Effects of Rubisco kinetics and Rubisco activation state on the temperature dependence of the photosynthetic rate in spinach leaves from contrasting growth temperatures. Plant, Cell & Environment 29, 1659–1670. 10.1111/j.1365-3040.2006.01550.x 16898026

[CIT0072] Zeder MA 2015 Core questions in domestication research. Proceedings of the National Academy of Sciences, USA 112, 3191–3198. 10.1073/pnas.1501711112 PMC4371924 25713127

[CIT0073] Zhu XG, Long SP, Ort DR 2010 Improving photosynthetic efficiency for greater yield. Annual Review of Plant Biology 61, 235–261. 10.1146/annurev-arplant-042809-112206 20192734

