# Supplementary material for: Leaf economics spectrum in rice: leaf anatomical, biochemical, and physiological trait trade-offs
Source: J Exp Bot. 2018 Sep 5;69(22):5599–609. doi: 10.1093/jxb/ery322 (PMC6255696; doi:10.1093/jxb/ery322)
Supplement: Supplementary Material [file ery322_suppl_supplementary_material.pdf]

The leaf economics spectrum in rice: leaf anatomical, biochemical and physiological traits trade-offs. Dongliang Xiong & Jaume Flexas

Supplementary data

Table S1. List of leaf traits in this study, and symbols and units adopted.

	Symbol	Trait	Unit
Function	A	Light-saturated photosynthetic rate	μmol m^{-2} s^{-1}
	g_s	Stomatal conductance to CO_2	mol m^{-2} s^{-1}
	g_m	Mesophyll conductance	mol m^{-2} s^{-1}
	K_{leaf}	Leaf hydraulic conductance	mmol m^{-2} s^{-1} MPa^{-1}
	V_{cmax}	Maximum carboxylation efficiency	μmol m^{-2} s^{-1} μmol
	J_{max}	Maximum rate of electron transport	m^{-2} s^{-1}
	A_m	Light-saturated photosynthetic rate per leaf mass	nmol g^{-1} s^{-1}
	g_{sm}	Stomatal conductance to CO_2 per leaf mass	nmol kg^{-1} s^{-1}
	g_{mm}	Mesophyll conductance per leaf mass	nmol kg^{-1} s^{-1}
	K_{leafm}	Leaf hydraulic conductance	mmol g^{-1} s^{-1} MPa^{-1}
Biochemistry	N_a	Nitrogen concentration per leaf area	g m^{-2}
	N_m	Nitrogen concentration per leaf mass	%
	Rubisco	Rubisco concentration per leaf area	g m^{-2}
	$Rubisco_m$	Rubisco concentration per leaf mass	g g^{-1}
Anatomy	LMA	Leaf mass per leaf area	g m^{-2}
	VLA	Leaf vein length per leaf area	mm mm^{-2}
	VLA_m	Leaf vein length per leaf mass	mm mg^{-1}
	LT	Leaf thickness	mm
	LD	Leaf density	mg mm^{-3}
	f_{IAS}	Fraction of leaf mesophyll volume occupied by intercellular air space	%
	T_{cw}	Cell wall thickness	μm
	S_m	Total mesophyll cell surface area exposed to intercellular air space per unit of leaf surface area	m^{-2} m^{-2}
	S_c	Total chloroplasts surface area exposed to intercellular air space per unit of leaf surface area	m^{-2} m^{-2}
Efficiency	PNUE	Photosynthetic N use efficiency	μmol g^{-1} (N)

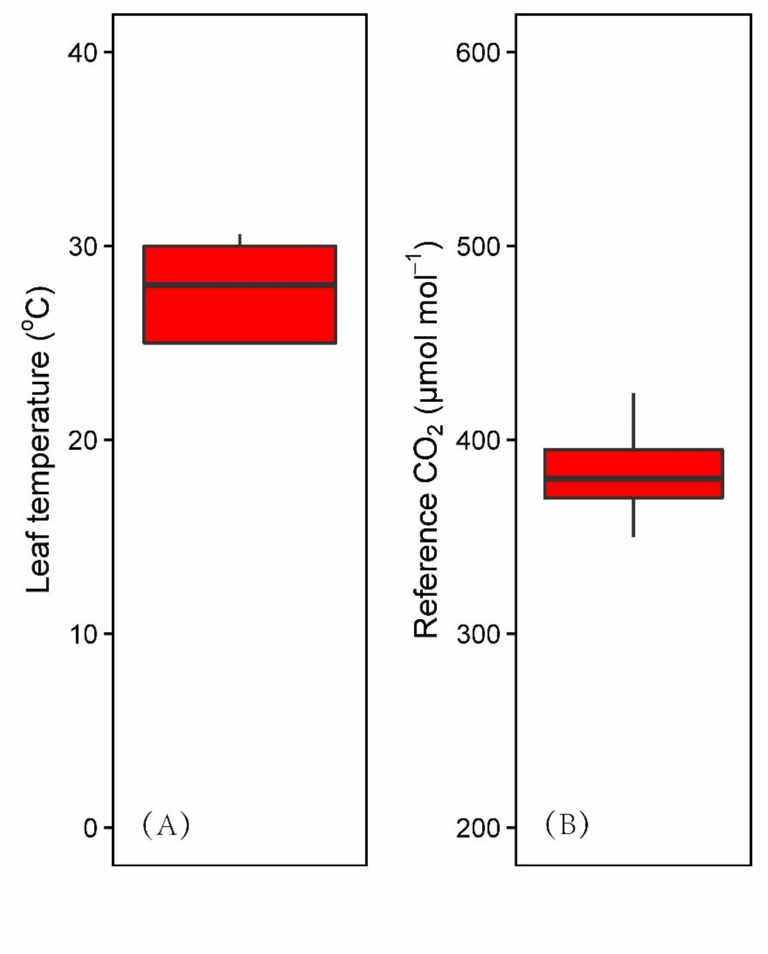

Figure S1 Leaf temperature (A) and the reference CO_2 concentration inside cuvette (B) for gas exchange measurements.

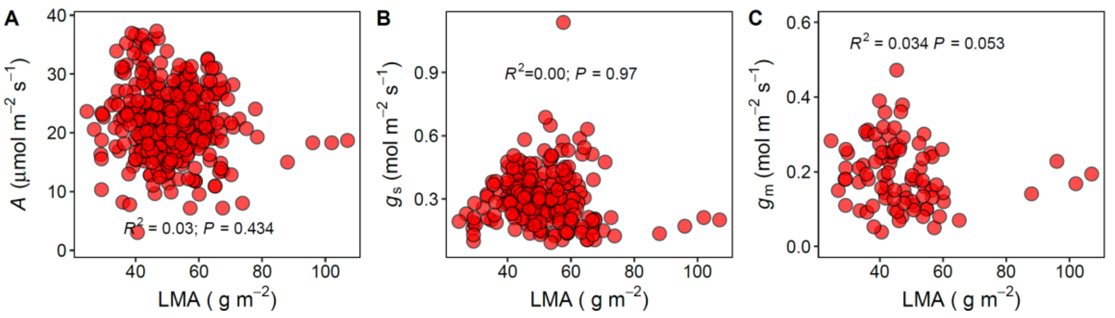

Fig. S2 Influences of the leaf mass per area (LMA) on the light-saturated photosynthetic rate (*A*), the stomatal conductance (g_s) and the mesophyll conductance (g_m).

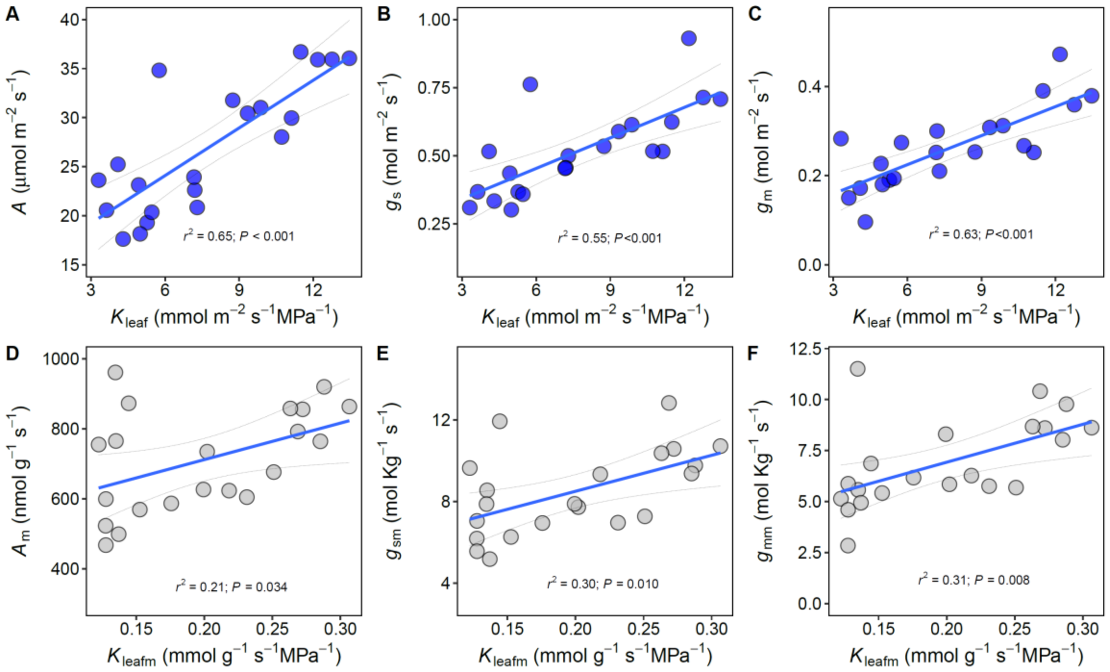

Figure S3. Correlations of the leaf hydraulic conductance (K_{leaf}) to (A, D) the light-saturated photosynthetic rate (A), (B, E) the stomatal conductance (g_s) and (C, F) the mesophyll conductance (g_m) in rice. A-C in the area-base and D-F in the mass-base. The shaded area indicates the 95% CI of the fitting.

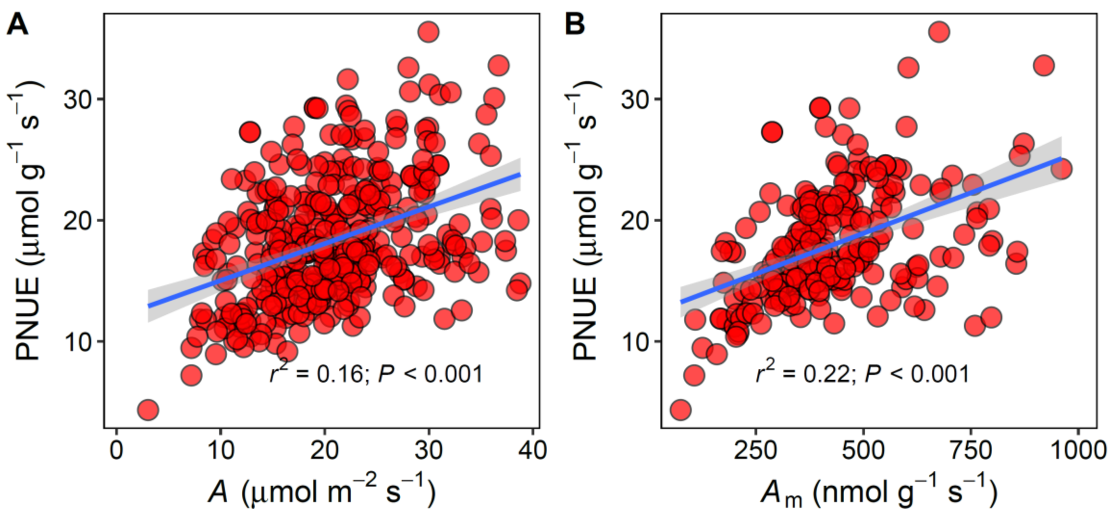

Figure S4. Correlations (A) between the area-based light-saturated photosynthetic rate (*A*) and the photosynthetic N use efficiency (PNUE); and (B) between the mass-based light-saturated photosynthetic rate (A_m) and the PNUE.

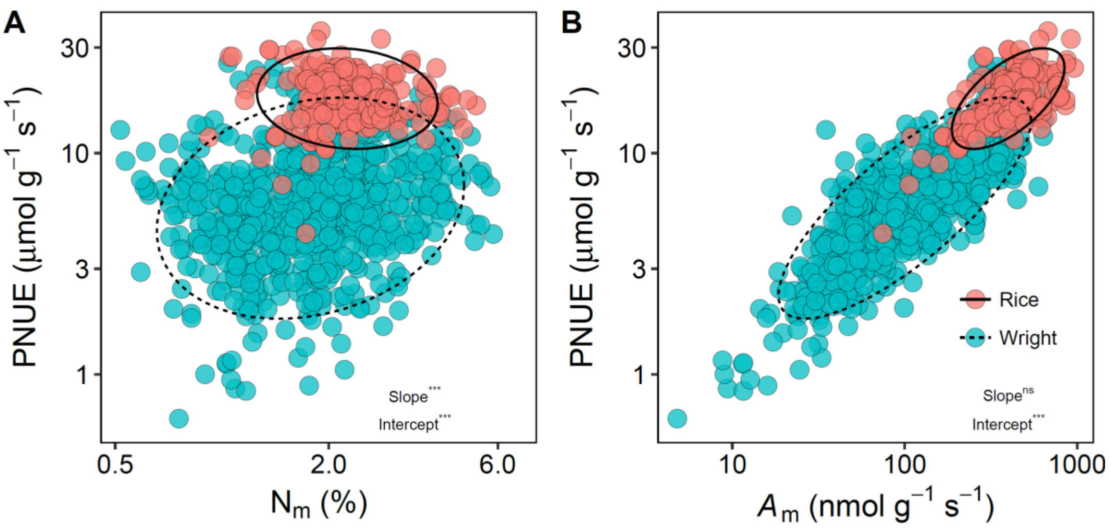

Figure S5. Effects of (A) the N concentration per leaf mass (N_m) and the mass-based light-saturated photosynthetic rate (A_m) on the photosynthetic N use efficiency (PNUE). Green circles represent the data from Glopnet (Wright *et al*., 2004) and red circles represent rice.

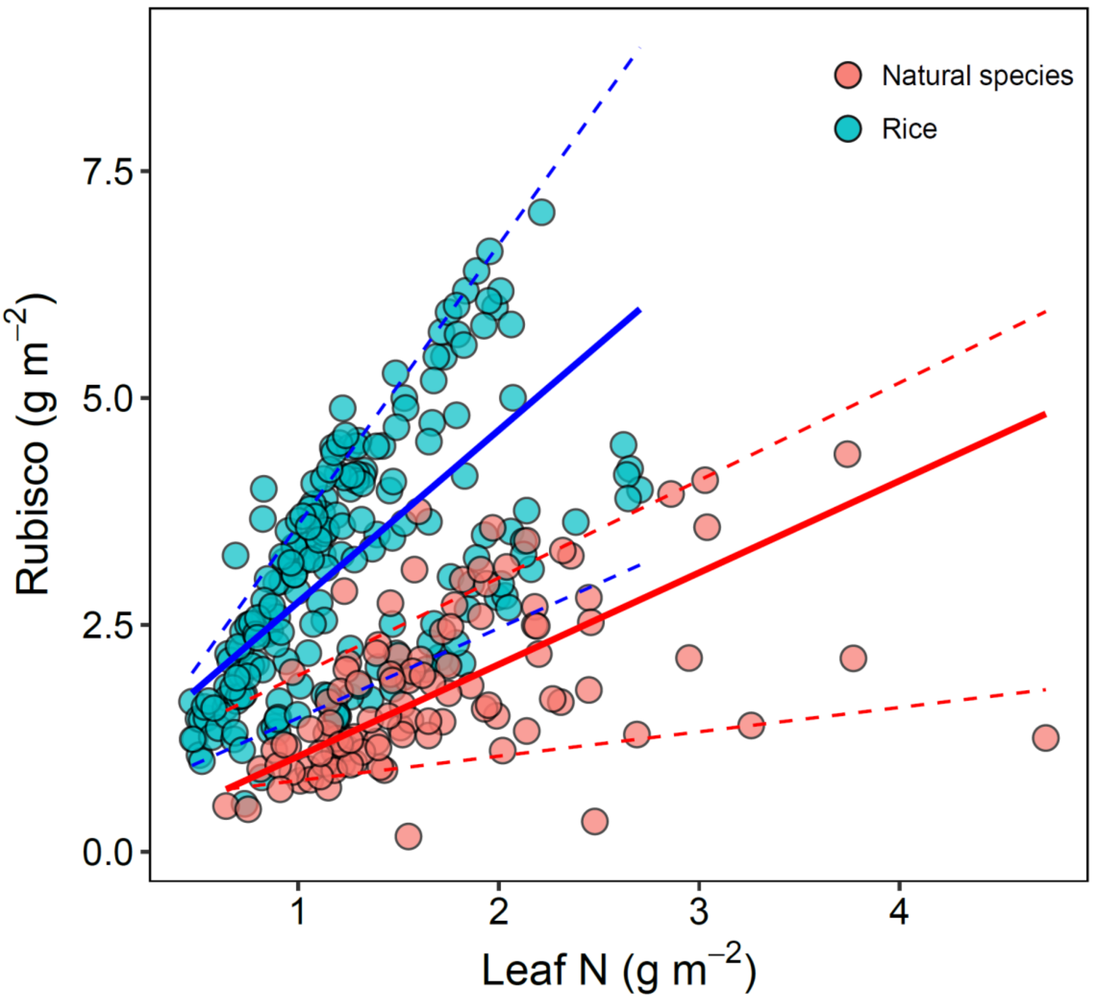

Figure S6. The correlation between Rubisco content and leaf N content within rice or among natural species (data from Onoda *et al.*, 2017). The lines were fitted using quantile regression model. Sold lines represent the quantile of 0.5 and dot lines represent the quantiles of 0.1 and 0.9.

Literature used in this study

1. **Adachi M, Hasegawa T, Fukayama H, Tokida T, Sakai H, Matsunami T, Nakamura H, Sameshima R, Okada M**. 2014a. Soil and water warming accelerates phenology and down-regulation of leaf photosynthesis of rice plants grown under free-air CO_2 enrichment (FACE). *Plant and Cell Physiology* **55**, 370-380.
2. **Adachi S, Baptista LZ, Sueyoshi T, Murata K, Yamamoto T, Ebitani T, Ookawa T, Hirasawa T**. 2014b. Introgression of two chromosome regions for leaf photosynthesis from an indica rice into the genetic background of a japonica rice. *Journal of Experimental Botany* **65**, 2049-2056.
3. **Adachi S, Nakae T, Uchida M, Soda K, Takai T, Oi T, Yamamoto T, Ookawa T, Miyake H, Yano M, Hirasawa T**. 2013. The mesophyll anatomy enhancing CO_2 diffusion is a key trait for improving rice photosynthesis. *Journal of Experimental Botany* **64**, 1061-1072.
4. **Giuliani R, Koteyeva N, Voznesenskaya E, Evans MA, Cousins AB, Edwards GE**. 2013. Coordination of leaf photosynthesis, transpiration, and structural traits in rice and wild relatives (*Genus Oryza*). *Plant Physiology* **162**, 1632-1651.
5. **Gong HY, Li Y, Fang G, Hu DH, Jin WB, Wang ZH, Li YS**. 2015. Transgenic rice expressing *Ictb* and *FBP/Sbpase* derived from cyanobacteria exhibits enhanced photosynthesis and mesophyll conductance to CO_2. *Plos One* **10**, e0140928.
6. **Guo S, Chen G, Zhou Y, Shen Q. 2007.** Ammonium nutrition increases photosynthesis rate under water stress at early development stage of rice (Oryza sativa L.). *Plant and Soil* **296**(1-2): 115-124.
7. **Harmens H, Marriott DJ, Ashenden TW**. 2005. Photosynthetic capacity and productivity of CO_2-enriched rice (*Oryza sativa* L.) under field conditions. *Journal of Crop Improvement* **13**, 55-72.
8. **Hirasawa T, Ozawa S, Taylaran RD, Ookawa T**. 2010. Varietal differences in photosynthetic rates in rice plants, with special reference to the nitrogen content of leaves. *Plant Production Science* **13**, 53-57.
9. **Huang M, Shan S, Zhou X, Chen J, Cao F, Jiang L, Zou Y**. 2016. Leaf photosynthetic performance related to higher radiation use efficiency and grain yield in hybrid rice. *Field Crops Research* **193**, 87-93.
10. **Huang G., Zhang Q., Wei X., Peng S. & Li Y.** 2017 Nitrogen Can Alleviate the Inhibition of Photosynthesis Caused by High Temperature Stress under Both Steady-State and Flecked Irradiance. Front Plant Sci, **8**, 945.
11. **Kanemura T, Homma K, Ohsumi A, Shiraiwa T, Horie T**. 2007. Evaluation of genotypic variation in leaf photosynthetic rate and its associated factors by using rice diversity research set of germplasm. *Photosynthesis Research* **94**, 23-30.
12. **Kumagai E, Araki T, Kubota F**. 2007. Effects of nitrogen supply restriction on gas exchange and photosystem 2 function in flag leaves of a traditional low-yield cultivar and a recently improved high-yield cultivar of rice (*Oryza sativa* L.). *Photosynthetica* **45**, 489-495.
13. **Kumagai E, Araki T, Kubota F**. 2009. Characteristics of gas exchange and chlorophyll fluorescence during senescence of flag leaf in different rice (*Oryza sativa* L.) cultivars grown under nitrogen-deficient condition. *Plant Production Science* **12**, 285-292.
14. **Li Y, Gao YX, Xu XM, Shen QR, Guo SW**. 2009. Light-saturated photosynthetic rate in high-nitrogen rice (*Oryza sativa* L.) leaves is related to chloroplastic CO_2 concentration. *Journal of Experimental Botany* **60**, 2351-2360.

15. **Li Y, Ren B, Ding L, Shen Q, Peng S, Guo S**. 2013. Does chloroplast size influence photosynthetic nitrogen use efficiency? *Plos One* **8**, e62036.
16. **Li Y, Ren B, Yang X, Xu G, Shen Q, Guo S**. 2012a. Chloroplast downsizing under nitrate nutrition restrained mesophyll conductance and photosynthesis in rice (*Oryza sativa* L.) under drought conditions. *Plant and Cell Physiology* **53**, 892-900.
17. **Li Y, Yang X, Ren B, Shen QR, Guo SW**. 2012b. Why nitrogen use efficiency decreased under high nitrogen supply of rice seedlings (*Oryza sativa* L.)? *Journal of Plant Growth Regulation* **31**: 47-52.
18. **Liu X, Li Y**. 2015. Varietal difference in the correlation between leaf nitrogen content and photosynthesis in rice (*Oryza sativa* L.) plants is related to specific leaf weight. *Journal of Integrative Agriculture* **15**, 2002-2011.
19. **Makino A, Nakano H, Mae T. 1994a.** Responses of ribulose-1,5-bisphosphate carboxylase, cytochrome f, and sucrose synthesis enzymes in rice leaves to leaf nitrogen and their relationships to photosynthesis. *Plant Physiol* **105**(1): 173-179.
20. **Makino A, Nakano H, Mae T. 1994b.** Effects of growth temperature on the responses of ribulose-1,5-bisphosphate carboxylase, electron transport components, and sucrose synthesis enzymes to leaf nitrogen in rice, and their relationships to photosynthesis. *Plant Physiol* **105**(4): 1231-1238.
21. **Makino A, Sato T, Nakano H, Mae T. 1997a.** Leaf photosynthesis, plant growth and nitrogen allocation in rice under different irradiances. *Planta* **203**(3): 390-398.
22. **Makino A, Shimada T, Takumi S, Kaneko K, Matsuoka M, Shimamoto K, Nakano H, Miyao-Tokutomi M, Mae T, Yamamoto N**. 1997b. Does decrease in Ribulose-1,5-Bisphosphate Carboxylase by antisense *RbcS* lead to a higher N-use efficiency of photosynthesis under conditions of saturating CO_2 and light in rice plants? *Plant Physiology* **114**, 483-491.
23. **Nakano H, Makino A, Mae T**. 1997. The effect of elevated partial pressures of CO_2 on the relationship between photosynthetic capacity and N content in rice leaves. *Plant Physiology* **115**, 191-198.
24. **Ohsumi A, Hamasaki A, Nakagawa H, Yoshida H, Shiraiwa T, Horie T**. 2007. A model explaining genotypic and ontogenetic variation of leaf photosynthetic rate in rice (*Oryza sativa*) based on leaf nitrogen content and stomatal conductance. *Annals of Botany* **99**, 265-273.
25. **Resurreccion A, Makino A, Bennett J, Mae T.** 2001 Effects of sulfur nutrition on the growth and photosynthesis of rice, *Soil Science and Plant Nutrition*, **47**, 611-620
26. **Scafaro AP, Von Caemmerer S, Evans JR, Atwell BJ**. 2011. Temperature response of mesophyll conductance in cultivated and wild *Oryza* species with contrasting mesophyll cell wall thickness. *Plant, Cell and Environment* **34**, 1999-2008.
27. **Seneweera S, Makino A, Hirotsu N, Norton R, Suzuki Y**. 2011. New insight into photosynthetic acclimation to elevated CO_2: The role of leaf nitrogen and ribulose-1,5-bisphosphate carboxylase/oxygenase content in rice leaves. *Environmental and Experimental Botany* **71**, 128-136.
28. **Shimono H, Suzuki K, Aoki K, Hasegawa T, Okada M.** 2010 Effect of panicle removal on photosynthetic acclimation under elevated CO_2 in rice. Photosynthetica **48**, 530-536
29. **Shimoda S, Maruyama A. 2014.** Rice varietal differences in responses of stomatal gas exchange to supplemental nitrogen application. *Photosynthetica* **52**(3): 397-403.

30. **Sudo E, Makino A, Mae T**. 2003. Differences between rice and wheat in ribulose-1,5-bisphosphate regeneration capacity per unit of leaf-N content. *Plant, Cell and Environment* **26**, 255-263.
31. **Sun, J, Ye M, Peng S, Li Y.** 2016 Nitrogen can improve the rapid response of photosynthesis to changing irradiance in rice (*Oryza sativa* L.) plants. *Scitific Reports* **6**, 31305
32. **Suzuki Y, Miyamoto T, Yoshizawa R, Mae T, Makino A**. 2009. Rubisco content and photosynthesis of leaves at different positions in transgenic rice with an overexpression of RBCS. *Plant, Cell and Environment* **32**, 417-427.
33. **Suzuki Y, Ohkubo M, Hatakeyama H, Ohashi K, Yoshizawa R, Kojima S, Hayakawa R, Yamaya T, Mae T, Makino A.** 2007 Increased Rubisco content in transgenic rice transformed with the 'Sense' *rbcS* gene *Plant Cell & Physiology* **48**, 626-637.
34. **Taylaran RD, Adachi S, Ookawa T, Usuda H, Hirasawa T**. 2011. Hydraulic conductance as well as nitrogen accumulation plays a role in the higher rate of leaf photosynthesis of the most productive variety of rice in Japan. *Journal of Experimental Botany* **62**, 4067-4077.
35. **Urairi C, Tanaka Y, Hirooka Y, Homma K, Xu Z, Shiraiwa T**. 2016. Response of the leaf photosynthetic rate to available nitrogen in erect panicle-type rice (*Oryza sativa* L.) cultivar, Shennong265. *Plant Production Science* **19**, 420-426.
36. **Xiong D, Flexas J, Yu T, Peng S, Huang J**. 2016a. Leaf anatomy mediates coordination of leaf hydraulic conductance and mesophyll conductance to CO_2 in *Oryza*. *New Phytologist*, 10.1111/nph.14186.
37. **Xiong D, Liu X, Liu L, Douthe C, Li Y, Peng S, Huang J**. 2015a. Rapid responses of mesophyll conductance to changes of CO_2 concentration, temperature and irradiance are affected by N supplements in rice. *Plant, Cell and Environment* **38**, 2541-2550.
38. **Xiong D, Wang D, Liu X, Peng S, Huang J, Li Y**. 2016b. Leaf density explains variation in leaf mass per area in rice between cultivars and nitrogen treatments. *Annals of Botany* **117**, 963-971.
39. **Xiong D, Yu T, Ling X, Fahad S, Peng S, Li Y, Huang J**. 2015b. Sufficient leaf transpiration and nonstructural carbohydrates are beneficial for high-temperature tolerance in three rice (*Oryza sativa*) cultivars and two nitrogen treatments. *Functional Plant Biology* **42**, 347-356.
40. **Xiong D, Yu T, Liu X, Li Y, Peng S, Huang J**. 2015c. Heterogeneity of photosynthesis within leaves is associated with alteration of leaf structural features and leaf N content per leaf area in rice. *Functional Plant Biology* **42**, 687-696.
41. **Xiong D, Yu T, Zhang T, Li Y, Peng S, Huang J**. 2015d. Leaf hydraulic conductance is coordinated with leaf morpho-anatomical traits and nitrogen status in the genus *Oryza*. *Journal of Experimental Botany* **66**, 741-748.
42. **Xiong D., Douthe C. & Flexas J.** 2018 Differential coordination of stomatal conductance, mesophyll conductance, and leaf hydraulic conductance in response to changing light across species. Plant, Cell and Environment, **41**, 436-450.
43. **Xue W, Nay-Htoon B, Lindner S, Dubbert M, Otieno D, Ko J, Werner C, Tenhunen J**. 2016. Soil water availability and capacity of nitrogen accumulation influence variations of intrinsic water use efficiency in rice. *Journal of Plant Physiology* **193**, 26-36.
44. **Yamori W, Makino A, Shikanai T**. 2016. A physiological role of cyclic electron transport around photosystem I in sustaining photosynthesis under fluctuating light in rice. *Scientific Reports* **6**, 20147.

45. **Yamori W, Nagai T, Makino A. 2011a.** The rate-limiting step for CO_2 assimilation at different temperatures is influenced by the leaf nitrogen content in several C_3 crop species. *Plant, Cell and Environment* **34**(5): 764-777.
46. **Yamori W, Sakata N, Suzuki Y, Shikanai T, Makino A. 2011b.** Cyclic electron flow around photosystem i via chloroplast NAD(P)H dehydrogenase (NDH) complex performs a significant physiological role during photosynthesis and plant growth at low temperature in rice. *Plant Journal* **68**(6): 966-976.
47. **Ye M., Peng S., and Li Y**. 2018 Intraspecific variation in photosynthesis is positively 1 related to photosynthetic nitrogen use efficiency in rice (*Oryza sativa* L.) plants**.** *Photosynthetica* (in press)
48. **Yu H, Murchie EH, González-Carranza ZH, Pyke KA, Roberts JA**. 2015. Decreased photosynthesis in the *erect panicle 3* (*ep3*) mutant of rice is associated with reduced stomatal conductance and attenuated guard cell development. *Journal of Experimental Botany* **66**, 1543-1552.
49. **Zhao M, Ding Z, Lafitte R, Sacks E, Dimayuga G, Holt D.** 2010 Photosynthetic characteristics in *Oryza* species. *Photosynthetica* **48**, 234-240.
50. **Zhu C, Ziska L, Zhu J, Zeng Q, Xie Z, Tang H, Jia X, Hasegawa T**. 2012 The temporal and species dynamics of photosynthetic acclimation in flag leaves of rice (*Oryza sativa*) and wheat (*Triticum aestivum*) under elevated carbon dioxide. *Physiologia Plantarum* 145, 395–405
